# Variations in bifid superior transverse scapular ligament as a possible factor of suprascapular entrapment: an anatomical study

**DOI:** 10.1007/s00264-012-1618-2

**Published:** 2012-07-25

**Authors:** Michał Polguj, Kazimierz Jędrzejewski, Agata Majos, Mirosław Topol

**Affiliations:** 1 Department of Angiology, Chair of Anatomy, Medical University of Łódź, Narutowicza 60, Łódź, 90-136 Poland; 2 Department of Normal and Clinical Anatomy, Chair of Anatomy, Medical University of Łódź, Narutowicza 60, Łódź, 90-136 Poland; 3 Radiology Department, Medical University of Łódź, Kopcińskiego 22, 90-153 Łódź, Poland

## Abstract

**Purpose:**

The suprascapular region is the most common place for suprascapular nerve entrapment. The purpose of this research was to investigate the influence of the superior transverse scapular ligament (STSL) on the reduced space for suprascapular nerve passage at the suprascapular notch. Additionally, we precisely described other structures that seem important during arthroscopic and open procedures in this region.

**Methods:**

The study was carried out on 96 formalin-fixed cadaveric shoulders. After dissection of the suprascapular region the superior transverse scapular ligament was visualised. Measurements of the ligaments and area of the opening for the passage of the suprascapular nerve were taken using two independent methods: MultiScanBase v.14.02 software and electronic digimatic caliper.

**Results:**

In three of 96 dissected shoulders (3.1 %), a bifid superior transverse scapular ligament was discovered. Morphologically, two subtypes of bifid STSL were distinguished. The first has medially separate superior and inferior bands and a common lateral attachment. In the second subtype, independent anterior and posterior parts are fixed to the lateral border of the suprascapular notch with a common medial attachment. Singular STSL in 93 of 96 shoulders was found. The mean area of the suprascapular opening in the specimens with singular STSL was larger than in the first subtype of bifid STSL and in the second subtype of bifid STSL with anterior coracoscapular ligament.

**Conclusion:**

Knowledge of the variations of the superior transverse scapular ligament is important for better understanding of the possible anatomical conditions that can promote suprascapular nerve entrapment. Its morphology should also be taken into particular consideration during surgical and arthroscopic procedures.

## Introduction

The superior transverse scapular ligament (STSL) connects two borders of the suprascapular notch on the upper edge of the scapula. Through this opening passes the suprascapular nerve (SN). The suprascapular region is the most common place for suprascapular nerve entrapment [1, 2].

In the scientific literature, Kopell and Thompson's report from 1959 is always regarded as the first description of the suprascapular nerve entrapment syndrome. However, there is no doubt that André Thomas in 1936 was the first to describe and correctly interpret the mechanisms leading to the development of suprascapular nerve entrapment syndrome [3].

The clinical findings of this neuropathy are deep and diffused poorly localized pain, weakness of abduction and external rotation of the upper extremity, and atrophy of the supraspinatus and infraspinatus muscles. As nonspecific symptoms can result in late diagnosis and long, difficult treatment, all anatomical studies of this pathology are particularly important, especially in orthopaedics and neurology [1, 2, 4, 5]. It has been estimated that 0.4–2 % of superior extremity girdle pain is caused by suprascapular nerve entrapment [1, 2].

Variations in the superior transverse scapular ligament and suprascapular notch are the most recognized possible predisposing factors for suprascapular nerve entrapment [6]. There are several classifications of the suprascapular notch [6–13], but less attention has been paid to differences of the STSL morphological form [6, 12]. Nevertheless, it is still very important as, according to Ticker et al. [12], the bifid type of entity might be one of the possible causes of suprascapular nerve entrapment. Also, Alone et al. [14] describe suprascapular nerve entrapment in a patient with a bifid superior transverse scapular ligament. However, these observations were only macroscopic and not supported by any calculations. Our study, using a professional program to analyse images, represents the first quantitative study of the space below STSL, where the suprascapular nerve is travelling. To the best of our knowledge it is the first report on morphological variations of bifid STSL (including its division into two different subtypes) in the scientific literature.

This knowledge is especially important during arthroscopic procedures and other open procedures of the shoulder region [15, 16]. It enables better recognition and understanding of the topography of the suprascapular region. We also analysed the potential influence of bifid STSL on the reduced space for suprascapular nerve passage in comparison to a singular one.

## Materials and methods

The study was carried out on 96 formalin-fixed cadaveric shoulders (47 left and 49 right) in the Department of Anatomy, Medical University of Lodz. The research project was approved by the Bioethics Commission of the Medical University of Lodz (protocol no. RNN/12/10/KE). The age and sex of the donors were unknown. In cadavers, a long incision along the clavicle was performed. Then, the skin was separated from the shoulder and pectoralis major muscle. The clavicle was released from the pectoralis major muscle and from its proximal fixation to the sternum. The trapezius muscle was detached from the clavicle, acromion process and spine of the scapula. Support was placed under the cadaver in order to reveal the anterior aspect of the scapula. The suprascapular nerve and vessels were identified. Photographic documentation was obtained from a standardized position of the camera and scapulae. The scapulae were fixed with an adjustable clamp and ring stand at the same distance from the camera. Digital photographic documentation was processed in MultiScanBase v.14.02 software (Computer Scanning System II, Warsaw, Poland) to obtain the measurements of the STSL. The second complementary, but independent, approach was a classical method using an electronic digimatic caliper (Mitutoyo Company, Japan).

**Fig. 1:**
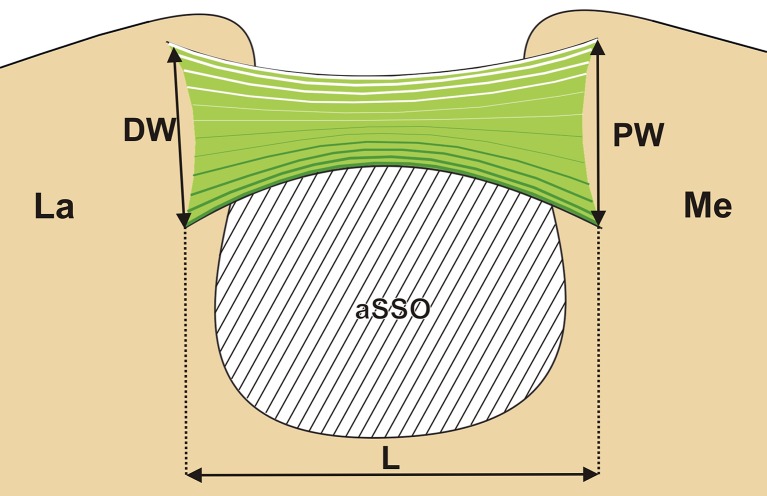
Schematic arrangements of the structures in the suprascapular region. Measurements of the superior transverse scapular ligament: *L* length, *PW* proximal width, *DW* distal width, *aSSO* area of the suprascapular opening. *La* lateral, *Me* medial

The following measurements of the structures at the suprascapular region were defined and performed (Fig. 1):

1. Length of the superior transverse scapular ligament (L)—the maximum value of the horizontal measurements taken in the horizontal plane between the proximal and distal boundaries of the ligament.
2. Proximal width (PW) of the superior transverse scapular ligament—the maximal value of the longitudinal measurements taken in the horizontal plane between the superior and inferior borders of the ligament at its proximal end.
3. Distal width (DW) of the superior transverse scapular ligament—the maximum value of the horizontal measurements taken in the horizontal plane between the superior and inferior borders of the ligament at its distal end.
4. Area of the suprascapular opening (aSSO)—the area limited superiorly by the inferior border of the superior transverse scapular ligament, laterally and medially by the osseous walls of the suprascapular notch, inferiorly by the superior border of the anterior coracoscapular ligament or inferior border of the SSN (specimens without anterior coracoscapular ligament).

## Results

**Fig. 2:**
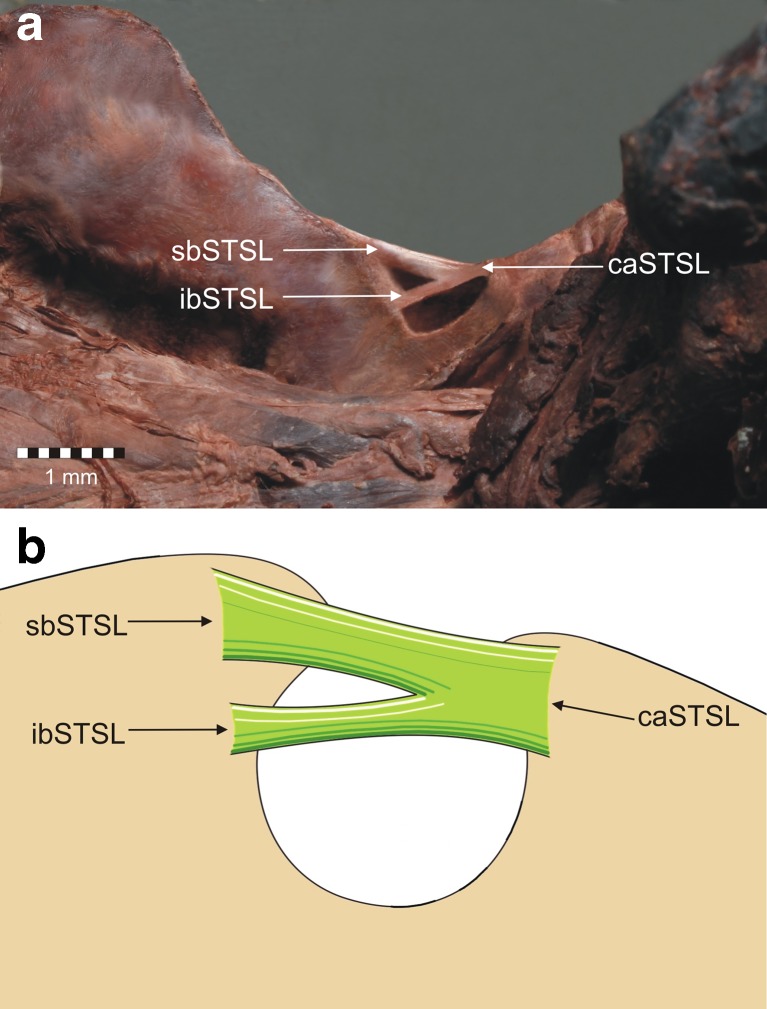
First type of the bifid superior transverse scapular ligament. **a** Structures at the cadaver. **b** Schematic arrangements. *sbSTSL* superior band of bifid superior transverse scapular ligament, *ibSTSL* inferior band of bifid superior transverse scapular ligament, *caSTSL* common attachment of bifid superior transverse scapular ligament

**Fig. 3:**
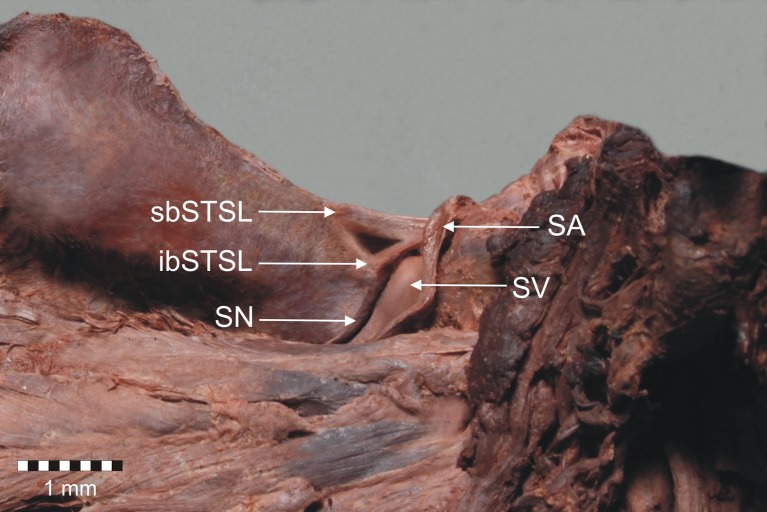
Anterior view of structures at the suprascapular region. *sbSTSL* superior band of bifid superior transverse scapular ligament, *ibSTSL* inferior band of bifid superior transverse scapular ligament, *SV* suprascapular vein, *SA* suprascapular artery, *SN* suprascapular nerve

**Fig. 4:**
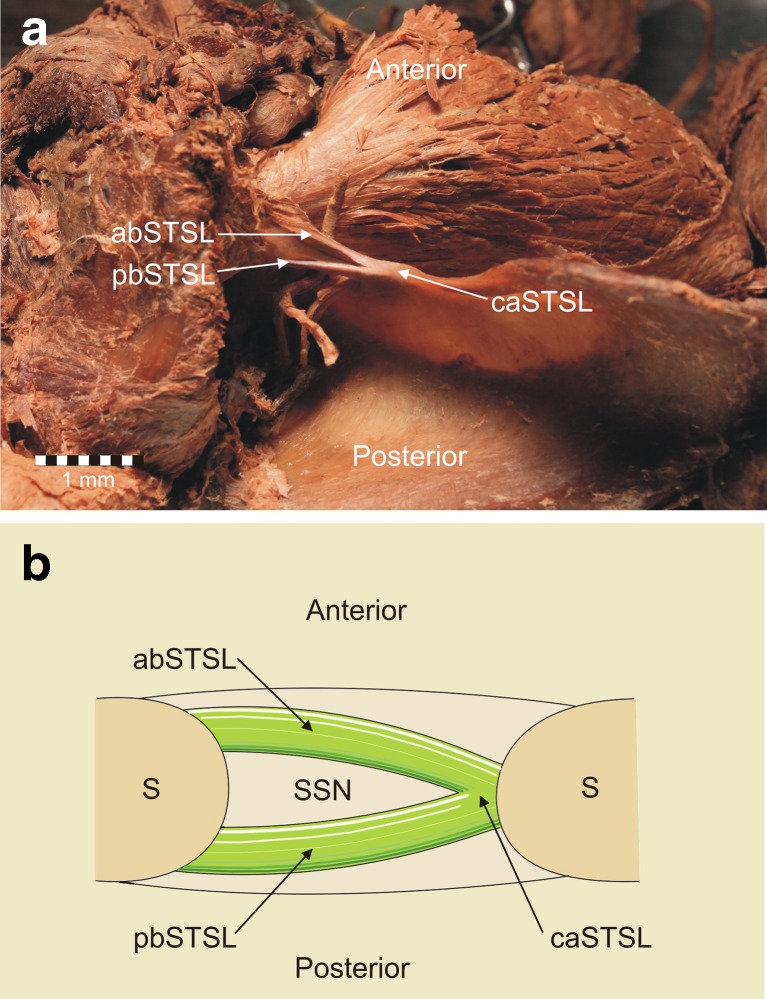
Second type of the bifid superior transverse scapular ligament (superior view). **a** Structures at the cadaver. **b** Schematic arrangements. *abSTSL* anterior band of bifid superior transverse scapular ligament, *pbSTSL* posterior band of bifid superior transverse scapular ligament, *caSTSL* common attachment of bifid superior transverse scapular ligament, *S* scapula, *SSN* suprascapular notch

In three of 96 dissected shoulders (3.1 %), a bifid superior transverse scapular ligament was discovered. According to the topography of the bifid STSL bands, morphologically, ligaments were divided into two subtypes. The first subtype has superior and inferior bands of the ligament (bifid in frontal plain) (Figs. 2, 3). In the second subtype of bifid STSL anterior and posterior bands were discovered (bifid in transverse plain) (Figs. 4, 6).

The first bifid STSL was found in the left upper extremity. The ligament had superior and inferior bands that separately fixed on the medial border of the suprascapular notch (Fig. 2). Both parts of this bifid STSL pass independently, one below the other (bifid in frontal plain), but laterally have a common attachment. Proximal and distal parts of the ligament were partially ossified. The suprascapular nerve and vein travel below the STSL, and the suprascapular artery above it (Fig. 3).

**Fig. 5:**
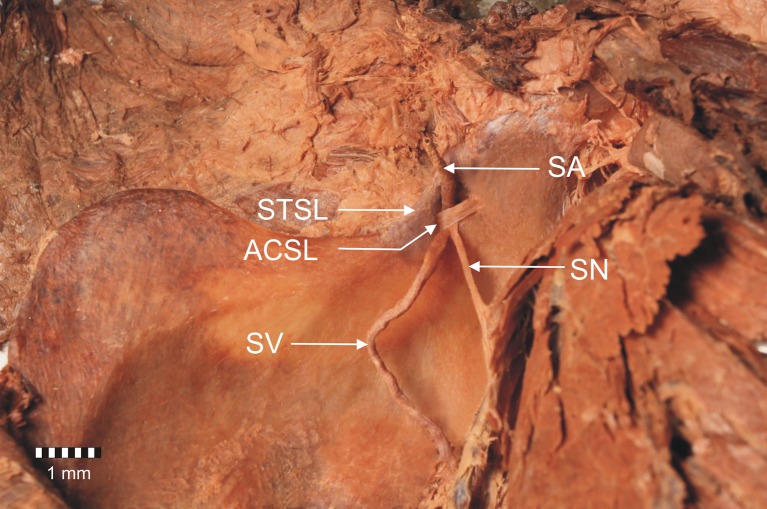
Anterior view of structures at the suprascapular region. *STSL* bifid superior transverse scapular ligament, *ACSL* anterior coracoscapular ligament, *SV* suprascapular vein, *SA* suprascapular artery, *SN* suprascapular nerve

**Fig. 6:**
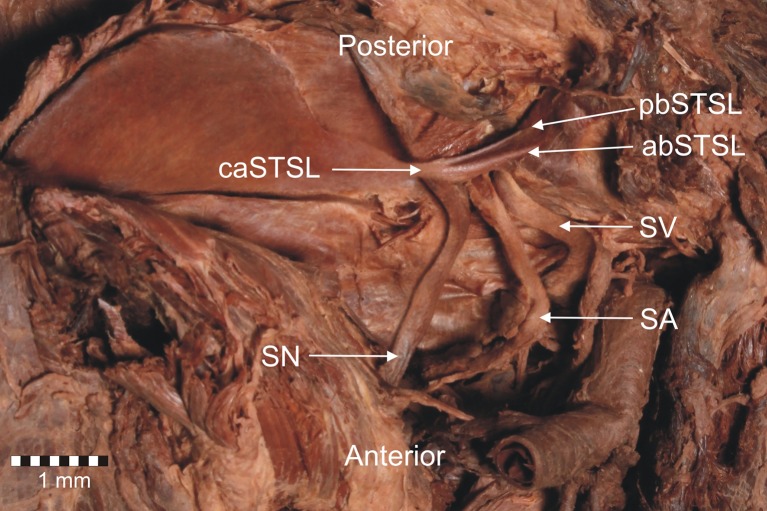
Anterior view of structures at the suprascapular region. *STSL* bifid superior transverse scapular ligament, *SV* suprascapular vein, *SA* suprascapular artery, *SN* suprascapular nerve

The second and third cases of bifid STSL were both found in the left upper limbs. The ligaments have anterior and posterior bands that were separately fixed to the lateral border of the suprascapular notch (Figs. 4, 6). Both parts of the bifid STSL travel one behind the other (bifid in the transverse plain). The bands were attached separately at the lateral border of the suprascapular notch and had a common medial attachment. (Figs. 4, 6). In the second cadaver with a bifid STSL the anterior coracoscapular ligament (ACSL) was found on the anterior aspect of the suprascapular notch below the STSL (Fig. 5). The suprascapular nerve, as well as the suprascapular vein, were situated below the ACSL (Fig. 5). The suprascapular artery passes between the STSL and ACSL (Fig. 5). In the third cadaver with a bifid superior transverse scapular ligament, the ACSL was absent. The suprascapular nerve and vein passed below both portions of the STSL, and the artery passed above them (Fig. 6).

**Fig. 7:**
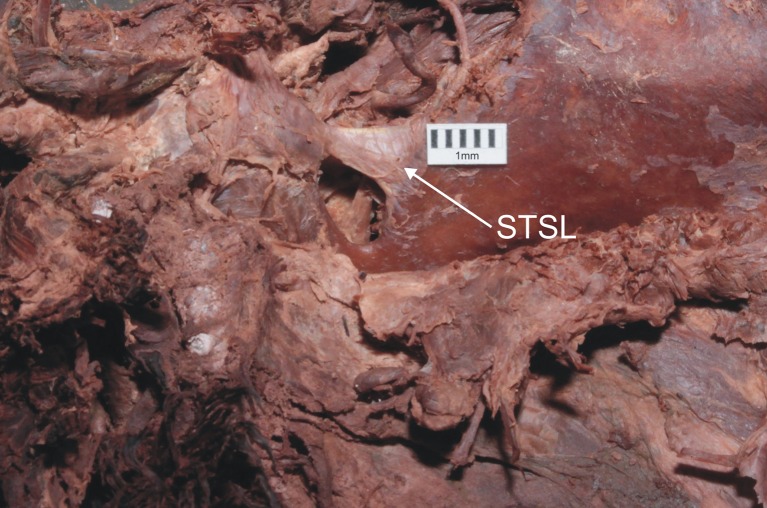
Anterior view of structures at the suprascapular region. *STSL* singular superior transverse scapular ligament

In 93 of 96 dissected shoulders (96.9 %) (44 left and 49 right), a singular superior transverse scapular ligament was discovered (Fig. 7). The anterior coracoscapular ligament was found in 47 of the 93 shoulders, on 24 right and 23 left, as an independent fibrous band on the anterior side of the suprascapular notch, below the STSL. In 91 cases the suprascapular nerve passed through the suprascapular foramen below STSL, while it passed inferior to the ACSL in two specimens. STSL was completely ossified in six of the shoulders studied.

According to our study the surface area of the suprascapular opening (aSSO) in the specimens with singular STSL amounted to 31.32 ± 18.92 mm^2^ (mean ± standard deviation) and was almost double that seen in the first subtype of bifid STSL (bifid in frontal plain)—19.40 mm^2^. Taking into consideration the second subtype of bifid STSL (bifid in transverse plain) the surface area of the suprascapular opening depended on presence of the anterior coracoscapular ligament. In the specimen with ACSL the area was smaller, 18.81 mm^2^ (second cadaver), and in the shoulder without ACSL it was larger 38.52 mm^2^ (third cadaver) in comparison to singular STSL.

The measurements of the STSL and the opening below this ligament are described for bifid STSL in Table 1 and for singular STSL in Table 2.

**Table 1:** Measurements of the bifid superior transverse scapular ligaments

Measurement		First cadaver		Second cadaver		Third cadaver	
		Superior portion	Inferior portion	Anterior portion	Posterior portion	Anterior portion	Posterior portion
Width (mm)	Proximal end	2.21	1.32	6.95		2.11	
	Distal end	1.61		2.73	2.13	2.52	2.10
Length (mm)		12.14	9.35	10.53	9.87	12.08	11.04
Area of SSO (mm^2^)		19.40		18.81		38.52	

*SSO* suprascapular opening

**Table 2:** Measurements of the singular superior transverse scapular ligaments

Measurement	Width (mm)		Length(mm)	Area of SSO (mm^2^)
	Proximal	Distal		
Mean	6.75	4.37	12.33	31.32
Standard	2.99	2.24	2.79	18.92
Deviation				
Minimum	1.35	1.2	6.55	4.6
Maximum	15.05	13.1	21.5	85.38
Mediana	6.15	3.85	12.05	25.03

*SSO* suprascapular opening

## Discussion

The literature has presented cases of multiband superior transverse scapular ligaments, a few bifid [6, 12, 14, 17] and one trifid [12].

A study by Ticker et al. [12] revealed that in two (3 %) of the 79 shoulders examined, multiband STSL were found. The bifid ligaments have two bands, anterior and posterior, which may be clinically important because a branch of the suprascapular nerve runs between them. They do not describe a bifid STSL with superior and inferior bands. The trifid STSL were found in a large “U” shaped suprascapular notch. The middle band was completely ossified. They do not describe ossification in bifid STSL.

Duparc et al. [17] note the presence of a double superior transverse ligament, with two different superposed bundles, in one shoulder (3.3 %). However, the STSL was single in 29 of 30 shoulders (96.7 %).

Bayramoglu et al. [6] describe five types of STSL. The first, most common, has a fan shape (53.1 %), while the second has an additional anterior coracoscapular ligament (18.8 %). The third type (15.6 %) consists of anterior and posterior parts, and the fourth, least common, type (12.5 %) of ligament is calcified. They do not describe a bifid STSL consisting of superior and anterior bands.

In our study, the frequency of the bifid superior transverse ligament was 3.1 %, which was similar to that seen by Duparc et al. [17] (3.3 %) and lower than that described by Bayramoglu et al. [6] (15.6 %). It would be reasonable to suppose that these frequencies depend on the population, like for example, the frequency of complete ossified STSL described below.

Probably one of the most important factors of suprascapular entrapment is the completely ossified superior traverse scapular ligament. The frequency of such cases varies throughout the world. In the European population, it varies according to country: 6.1 % in Italy (Vallois [18]), 6.5 % in France (Vallois [19]), 7 % in Poland (Polguj et al. [9]), and 7.3 % in Germany (Natsis et al. [8]). Complete ossification of the STSL in the US population was found in 3.7–5.5 % of cases (Rengachary et al. [10], Ticker et al. [12] and Avery et al. [20]). There were cases from another continents including Africa with 3 % of cases (Kenya—Sinkeet et al. [11]), Asia with 4.08 % of cases (China—Wang et al. [13]), which even saw 6.0–12.5 % of cases in Turkey (Urguden et al. [21], Bayramoglu et al. [6]). However, in some populations, complete ossification was very rare, such as the Alaskan Eskimos at 0.3 % [7]. In our study, we found six completely ossified STSL (6.3 %). Additionaly, partially ossified proximal and distal parts of the first subtype of bifid ligament (bifid in the frontal plane) was discovered.

Also very interesting is the study of Pecina et al. [22]. Scientists discovered that another factor of suprascapular nerve entrapment can be a connective tissue band, the ligamentum spinoglenoidale, which may exist in up to 50 % of people, creating a second fibro-osseous tunnel (spinoglenoid notch) for the nerve to transverse, and terminating in two, three or four motor branches that supply the infraspinatus muscle.

Another factor of suprascapular nerve entrapment is probably the anterior coracoscapular ligament first described by Avery et al. [20] in 2002 in 16 of 27 cadavers studied (60 %). Also Bayramoğlu et al. [6] confirmed the presence of an ACSL in six of the 32 shoulders (18.8 %) and proposed its presence as an additional etiological factor of the condition. Scientists propose that it might reduce the space available for the suprascapular nerve passage [6, 20]. In the present study, the frequency of the anterior coracoscapular ligament was 50 %. It was higher than that seen by Bayramoğlu et al. [6] (18.8 %) in the Turkish population and Piyawinijwong and Tantipoon [23] (28 %) in the Thais population, but was lower then that described by Avery et al. [20] (60 %) in the US population.

The superior transverse scapular ligament probably protects the suprascapular nerve but is also described as one of the important factors causing suprascapular nerve entrapment—especially the bifid type [14]; therefore, the knowledge of its morphology is useful, especially in orthopaedics. Arthroscopic techniques for suprascapular nerve decompression at the suprascapular notch have been recently described with successful results [15, 16, 24]. However, the safety and success of both arthroscopic and open procedures are predicated on a thorough understanding of the anatomy of the suprascapular region.

The first subtype of bifid STSL (bifid in frontal plain) can probably be a potential risk factor in suprascapular nerve entrapment because the space below the ligament through which the nerve passes is reduced in comparison to singular one. The area of suprascapular opening in this type (Table 1—first cadaver) was also twice as small than that in the second subtype of bifid STSL (bifid in transverse plain) (Table 1—third cadaver), respectively: 19.40 mm^2^ vs. 38.52 mm^2^. However, the problem is more complex, and in our opinion it depends on many factors such as shape of the suprascapular notch or presence of the anterior coracoscapular ligament. As described in our study, in the second cadaver with bifid STSL (Table 1), the anterior coracoscapular ligament can also narrow the space below the STSL and additionally compress the nerve in the suprascapular notch.

## Conclusion

Knowledge of the morphology of the suprascapular region is important for a better understanding of the possible anatomical conditions that can promote suprascapular nerve entrapment. Variations of the superior transverse scapular ligament should also be taken into particular consideration during surgical and arthroscopic procedures. The division of bifid superior transverse scapular ligament proposed in this study can be clinically important as the first type may well narrow the opening of the suprascapular notch.
